# Cell^3^: a new vision for study of the endomembrane system in mammalian cells

**DOI:** 10.1042/BSR20210850C

**Published:** 2021-12-07

**Authors:** Margaritha M. Mysior, Jeremy C. Simpson

**Affiliations:** Cell Screening Laboratory, UCD School of Biology and Environmental Science, University College Dublin, Dublin 4, Ireland

**Keywords:** 3D cell biology, endomembrane system, high-content imaging, spheroid, trafficking

## Abstract

The endomembrane system of mammalian cells provides massive capacity for the segregation of biochemical reactions into discrete locations. The individual organelles of the endomembrane system also require the ability to precisely transport material between these compartments in order to maintain cell homeostasis; this process is termed membrane traffic. For several decades, researchers have been systematically identifying and dissecting the molecular machinery that governs membrane trafficking pathways, with the overwhelming majority of these studies being carried out in cultured cells growing as monolayers. In recent years, a number of methodological innovations have provided the opportunity for cultured cells to be grown as 3-dimensional (3D) assemblies, for example as spheroids and organoids. These structures have the potential to better replicate the cellular environment found in tissues and present an exciting new opportunity for the study of cell function. In this mini-review, we summarize the main methods used to generate 3D cell models and highlight emerging studies that have started to use these models to study basic cellular processes. We also describe a number of pieces of work that potentially provide the basis for adaptation for deeper study of how membrane traffic is coordinated in multicellular assemblies. Finally, we comment on some of the technological challenges that still need to be overcome if 3D cell biology is to become a mainstream tool toward deepening our understanding of the endomembrane system in mammalian cells.

## Introduction

The cell can be defined as the basic building block of the tissues and organs that ultimately build the human body. The success of complex multicellular organisms has in no small part come as a consequence of not only the specialization of cell lineages but also through compartmentalization and the ability to spatially and temporally segregate biochemical reactions into different parts of the cell. In this regard, the endomembrane system, primarily comprising the endoplasmic reticulum (ER), Golgi apparatus, endosomes and lysosomes, plays a critical role. These organelles host a huge variety of macromolecules, which together play essential roles in processes such as protein and lipid synthesis, degradation, autophagy, and signaling. The endomembrane system can broadly be divided into three pathways: the anterograde pathway, the retrograde pathway and the endocytic pathway. The anterograde pathway is responsible for the synthesis and secretion of newly synthesized proteins, lipids and carbohydrates, from the ER via the Golgi apparatus, ultimately delivering material to various destinations [1]. The endocytic pathway is used for the cellular uptake of molecules and the sorting and recycling of plasma membrane-localized membrane receptors. Internalized material can be directed into the endosomal-lysosomal system, or in some cases it can be trafficked toward the Golgi apparatus and ultimately the ER via the retrograde pathway. Altogether, these pathways provide a convenient mechanism for the cell to be able to connect all the key compartments of the endomembrane system, they facilitate the recycling of trafficking machinery molecules back to their site of origin, and importantly they maintain the overall homeostasis of the cell. It is therefore unsurprising that any disruption to any one of these pathways is likely to have consequences on the others, as well as on the morphology and function of the participating organelles. Similarly, mutations or alterations in the levels of particular molecules can result in changes to organelle morphology and function, with consequences on the associated trafficking pathways [2]. Indeed, many diseases are linked to dysfunction of specific organelles such as the ER, Golgi and lysosomes [3–6]. For these reasons, the study of membrane traffic has for many years been of fundamental importance to our wider understanding of cell biology, not least because of the ever increasing links between endomembrane system dysfunction and disease [2].

To date, the overwhelming majority of studies addressing the molecular regulation of the endomembrane system have utilized conventional two-dimensional (2D) monolayer cells. Cells cultured in this format are easy to manipulate, they replicate quickly, are amenable to a wide range of perturbation types, and are compatible with both electron and light microscopy approaches. However, despite the massive advancements in our understanding of basic cell function, there remains the incongruity that cells rarely grow as simple monolayers in our tissues and organs. For decades we have ‘conveniently’ sidestepped the study of basic cell function in a three-dimensional (3D) context, despite the calls to do so [7,8], perhaps due to the many challenges associated with such experiments. Unsurprisingly, a number of studies have shown that when cells are grown in the form of 3D assemblies, therefore better mimicking the physiological environment, that there are differences in gene expression and their response to drugs. This is arguably of the greatest importance in the context of cancer research, drug delivery and toxicology, and it is in these research areas that we have seen 3D cell models being used more frequently [9–12]. 3D cell models can broadly be categorized into two groups, namely organoids and spheroids. Organoids are structures that mimic organs and are grown from adult stem cells, induced pluripotent stem cells, pluripotent stem cells or embryonic stem cells. They are predominantly utilized in disease modeling, developmental biology, drug toxicity testing and personalized medicine applications [13–15]. On the other hand, spheroids are assemblies of either one cell type or multiple cell types, which adopt various shapes. Spheroids can have a regular shape such as a round and mass morphology, or they can have an irregular shape, such as grape-like or stellate [16]. Many different types of cells are used to grow spheroids [17], and when cancer cells are used as the source of cells to generate spheroids then they are termed multicellular tumor spheroids (MCTS). These particular spheroids can begin to replicate the tumor environment with respect to oxygen and nutrient gradients, as well as the development of a necrotic core at their center [18]. Despite the increased utilization of 3D cell models in a number of research areas, there are comparatively few studies in which they have been used to study the endomembrane system. In this mini-review, we will assess why this is the case and provide a perspective on their importance and potential in furthering our understanding of how membrane trafficking is regulated throughout the endomembrane system.

## Overview of methods to produce 3D cell assemblies

**Figure 1:**
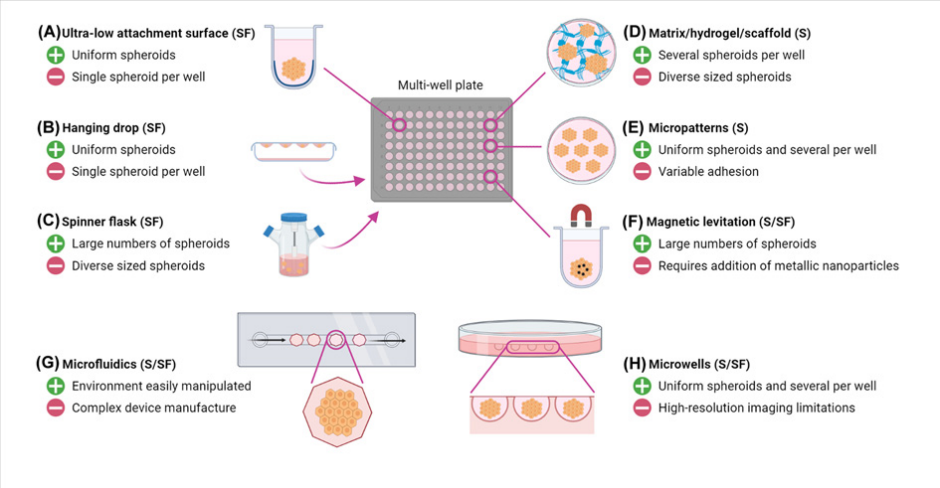
Main methods for production of spheroids Spheroids can be produced by a variety of scaffold (S) and scaffold-free methods (SF), depending on the cell type and required downstream application. Using a multi-well plate format, the commonly used methods include (**A**) ultra-low attachment surfaces, (**B**) hanging drop, (**C**) spinner flasks, (**D**) addition of an extracellular matrix material, (**E**) micropatterning and (**F**) magnetic levitation. Lines indicate that the spheroids can be prepared directly in the multi-well plate using this method, arrows indicate methods that require subsequent transfer of the spheroids into the multi-well plate after assembly. Other formats include (**G**) microfluidic devices and (**H**) microwells in a variety of dish types. For each method, one key advantage and disadvantage is given.

There are a variety of methods to prepare and culture 3D cell models, and these have been extensively discussed elsewhere [12,18–20]. Here we focus on methods that are likely to have the greatest applicability to study the endomembrane system of cells growing in 3D. One convenient way of categorizing these methods is to divide them into scaffold-free and scaffold-based approaches. Scaffold-free approaches create an environment that does not allow attachment of the cells to a substrate, effectively forcing the cells to aggregate and assemble into 3D structures. Probably the most popular of these has been the use of liquid overlay techniques, for example utilizing ultra-low attachment (ULA) plates, which have a surface to which cells cannot adhere. Other scaffold-free approaches include the hanging drop method, and continual rotation or agitation (Figure 1A–C). Magnetic levitation is a method that can work in the absence or presence of scaffolds (Figure 1F) [12,18–20]. By contrast, scaffold-based approaches utilize some type of matrix to provide physical support to the cells. These scaffolds can be made from various materials such as sponges, polymers, membranes and biocompatible inserts. The most popular materials are the natural extracellular matrix (ECM) mixture termed Matrigel [21], as well as other natural ECM products and synthetic hydrogel polymers (Figure 1D) [22]. Detailed descriptions of how each of these techniques can be employed are beyond the scope of this mini-review, and excellent articles can be found elsewhere [12,18–20]. Recently, the field has seen a number of innovations in terms of devices to facilitate the assembly and study of 3D cell assemblies. Each method has its particular advantages and disadvantages [20], and there are a number of technical considerations to keep in mind. For example, many of the commonly used scaffolds have particular handling requirements. Matrigel has two different aggregate states at 10°C (gel and liquid) and thus it can be challenging to use in automated or semi-automated cell assembly processes that involve robotics. Furthermore, the batch-to-batch variability of Matrigel can influence the growth of spheroids [23]. However, the advantage of many scaffold-based methods is that many spheroids can be grown per well. Scaffold-free technologies, such as U-bottomed ULA plates or the hanging drop method, have the disadvantage of producing only one spheroid per well, however have the advantage that the spheroids are usually uniform in size and shape [18,24]. As a general guide, spheroids can be assembled using both scaffold-free and scaffold-based methods, whereas organoids require the support of, and interactions with, the ECM. Also of note is that organoids typically require much longer growth times than spheroids, allowing them to fully develop. Important considerations for selecting the most appropriate 3D cell culture method include the size, number and consistency of the structures desired, as well as how they will be subsequently analyzed (Figure 1). This latter point is particularly relevant in the context of studying the endomembrane system, which has the specific requirement that the method needs to maintain the spatial identity of the organelles and membrane carriers under study. In this regard, optical microscopy has become a mainstay technique for study of membrane trafficking pathways, but issues such as light scattering present particular challenges when imaging living 3D models [25].

## Defining the need for creating 3D cell assemblies

It is the fields of cancer biology, drug delivery and toxicology that have been the most prolific in embracing 3D cell culture technologies. It is not surprising that these research areas can benefit from utilizing assemblies of cells, for example to study the ability of therapeutics to penetrate into a tumor-like mass. Also noteworthy, are the increasing number of reports indicating that results obtained from monolayer cultures can be significantly different from those obtained from 3D structures. Several studies have shown alterations in gene expression as well as differences in drug response and toxicity [26–31]. This clearly suggests that the physical arrangement of the cells, and the interactions that they make in the 3D environment, have the potential to elicit functional differences in cell behavior compared with when grown as monolayers. Given these observations, perhaps it is timely to consider in a broader sense, whether the morphological arrangements of cells can impact other cellular events and processes. In the context of membrane trafficking, perhaps the most obvious first site to examine is the plasma membrane, and the routes taken by cargo to reach this destination. This question becomes especially relevant in cell types such as epithelial cells, which possess a plasma membrane with differential functionality, providing both an apical and basolateral face. This polarity within an individual cell is vital for many processes and is controlled by an extensive regulatory network [32]. Study of polarity in epithelial cells has predominantly come from experimental systems, pioneered by the work of David Sabatini, which utilize semi-permeable membrane filters to support tightly opposed assemblies of cells [33]. Although this arrangement allows cells to form distinct apical and basolateral surfaces, the culture is effectively growing as a monolayer. While these culture conditions have undoubtedly revolutionized our molecular understanding of epithelial cell polarity, they are not a *bona fide* 3D system, and are not designed to recapitulate the morphological environment found in tissues. Therefore, many questions remain open with respect to how the endomembrane system is organized and regulated in the 3D environment. For example, do the organelles of the secretory pathway adopt different morphologies, positions and sizes compared with those seen in monolayer culture? Are the trafficking pathways regulated with the same molecular machinery in cells that are deep inside a large 3D assembly, versus those cells that are more peripheral? How do cells regulate the composition of their plasma membrane, given that they are making cell–cell interactions on all sides? Does attenuation of membrane trafficking pathways provide the mechanism to regulate cell size in a 3D structure? While answering such questions will be challenging, undoubtedly such insight will be of great value to deepen our understanding of cell function in both normal and healthy tissue, and may provide avenues for therapeutic intervention in a disease context.

## Membrane traffic studies in 3D

Arguably the first significant attempt to study membrane trafficking pathways in a 3D culture system was reported by Mrozowska and Fukuda in 2016 [34]. In this work, they investigated the transport of the apical marker podocalyxin in Madin-Darby Canine Kidney (MDCK) II epithelial cells grown as both monolayers and 3D ‘cysts’. These cysts were generated in Matrigel and could be visualized by fluorescence microscopy as a single layered spheroid, with a hollow lumen containing the apical membrane, and with the basolateral side oriented to the outside of the cyst. They employed co-localization and RNA interference (RNAi) approaches to systematically assess the distribution of podocalyxin, in the context of the Rab family of small GTPases, in monolayer cells and cysts. Interestingly, the study highlighted that the depletion of particular Rab proteins resulted in different phenotypes in the 2D versus 3D cell models. For example, the knockdown of Rab13 and Rab14 caused an inhibition of podocalyxin transport to the apical plasma membrane in monolayer cells, whereas it was the knockdown of Rab4a/b, Rab15, Rab19 and Rab25 that caused an inhibition of podocalyxin transport in spheroids. One specific output from this study was the realization that different effectors of Rab35 seem to be differentially involved in the regulation of podocalyxin transport between 2D and 3D models. More recent work from the same group has focused on a number of the guanine nucleotide exchange factors (GEFs) that regulate Rab35 and have found that two of these, DENN domain-containing 1A and folliculin seem to be the specific molecules that influence the activity of Rab35 [35]. Unlike the findings in several of the drug delivery studies mentioned above, the differences in podocalyxin behavior between the 2D and 3D models were not found to occur as a result of changes in gene expression levels of the GEFs but rather a consequence of the subcellular localization of the GEF proteins themselves. Given the complexity of the endomembrane system in cells, it is highly likely that such a mechanism to regulate different behaviors in 2D and 3D is a more widespread phenomenon. While the MDCK II cyst model might be considered relatively simple in terms of its level of 3D complexity, it nevertheless also provides fascinating insight into how membrane traffic machinery is instrumental in driving the morphological arrangement of 3D structures, and specifically the formation of the central hollow lumen [34]. Of course one problem with the MDCK II cyst model is the orientation of the apical plasma membrane on the inside of the structure, thereby making uptake studies of exogenous cargoes somewhat challenging. Recently however, a protocol that causes the inversion of the spheroids (in this particular case intestinal spheroids or enteroids) was reported [36]. Remarkably, this inversion of the apical and basolateral membranes from the initial inside-out configuration was possible after formation of the original hollow structure and was driven by cell culture medium changes and accessibility of the cells to ECM components. This model was then used to assess the interactions and uptake of pathogenic *Salmonella* sp. but holds great potential for studying the uptake of a wide range of molecules.

Work in our own laboratory has sought to investigate the membrane trafficking machinery regulating endocytosis of nanocarriers, as potential therapeutic vehicles, again in an attempt to identify commonalities and differences between 2D and 3D cell models. RNAi screening of approximately 400 genes associated with membrane traffic and cytoskeleton regulation was initially used to define the key machinery associated with delivery of polymeric nanoparticles to lysosomes. Much of this work was also focused on the Rab GTPases and identified Rab33b as being a critical regulator of nanoparticle trafficking to lysosomes in monolayer cells [37]. Subsequently, we repeated this RNAi approach, albeit on a pilot scale, but using a solid spheroid model of HT-29 adenocarcinoma cells [38]. Interestingly, while Rab33b also proved to be a strong regulator of this process in the spheroids, depletion of the endocytic regulator Rab5a was found not to severely impact delivery of the nanoparticles to lysosomes in this model, contrary to our findings in monolayer cells. While this spheroid study was small-scale, it again points to the fact that there are differences in the mechanics of the endomembrane system depending on the growth format of the cells. Importantly, this study also demonstrated that high-resolution fluorescence data, at the subcellular level, could be extracted and quantified from such spheroids. Others have also used 3D cell models to measure delivery efficacy of nanoparticles synthesized from a variety of materials into cells. These studies have shown that uptake and penetration were dependent on the size and charge of the nanoparticle, and that the process could be inhibited by chemical inhibitors of endocytosis [39–42]. Unfortunately, these studies do not present subcellular resolution information, which clearly would be invaluable to reveal the membrane trafficking pathways that these nano-sized objects take, not only within individual cells, but between neighboring cells within the spheroid. Such information would undoubtedly be helpful for the design of nanomedicines. All of the above studies are summarized in Table 1.

**Table 1:** Summary of membrane trafficking studies in 3D

Cell type	3D cell culture method	3D cell model	Cellular process investigated	Imaging and analysis approach	Reference
MDCK II cells	Matrigel	Cyst	Regulation of podocalyxin trafficking by Rab GTPases to apical membranes	Manual point-scanning confocal microscopy, single plane analysis	Mrozowska and Fukuda, 2016 [34]
MDCK II cells	Matrigel	Cyst	Rab35 regulation of podocalyxin trafficking to apical membranes	Manual point-scanning confocal microscopy, single plane analysis	Kinoshita et al., 2020 [35]
HT-29 cells	Matrigel	Spheroid	Endocytosis of nanoparticles	Automated high-content spinning disk confocal microscopy, single plane analysis	Cutrona and Simpson, 2019 [38]
BxPC-3, PANC-1 and NIH3T3 cells	U-bottomed ULA plates	Spheroid	Endocytosis of nanoparticles	Manual point-scanning confocal microscopy, single plane analysis	Durymanov et al., 2019 [39]
AsPC-1	Hanging drop plates	Spheroid	Endocytosis of nanoparticles	Manual point-scanning confocal microscopy, single plane analysis	Lu et al., 2015 [40]
HCT-116 cells	U-bottomed ULA plates	Spheroid	Endocytosis of nanoparticles	Manual point-scanning confocal microscopy, single plane analysis	Tchoryk et al., 2019 [41]
BxPC-3 cells	Hanging drop plates	Spheroid	Endocytosis of nanoparticles	Manual point-scanning confocal microscopy, single plane analysis	Wang et al., 2020 [42]

Summary of studies in which 3D cell culture systems has been used to study aspects of membrane trafficking. All are detailed in the main text.

## Emergence of novel methodologies

One significant challenge of working with 3D cell models is selecting a growth method that finds the best compromise between providing a physiologically relevant assembly, and which is amenable to their study, for example using high-resolution imaging. A variety of fluorescence microscopy modalities are potentially suitable for imaging 3D assemblies, including confocal microscopy, light-sheet microscopy, super-resolution microscopy and multi-photon microscopy. These techniques can be combined with advanced clearing protocols to minimize light scattering [43]. Another emerging and exciting option, which can be combined with the above techniques, is expansion microscopy. This technique utilizes incubation with polymers to enable the physical expansion of the spheroid, organoid or tissue, and thus increase the size of the cells without affecting the morphology of the 3D environment [44,45]. Common to many of the works described above has been the use of Matrigel as the ECM material to facilitate 3D assembly formation. Although Matrigel is widely used for support of spheroid growth, it does present challenges in its handling, as discussed above. However, it has found favor due its high cell-compatibility as well as showing minimal optical interference with fluorescence microscopy techniques. Recently, a technique was reported that describes the assembly of mini spheroids of MCF10A breast cancer epithelial cells, using an automated liquid handling system, in drops of just 0.2 µl of Matrigel [46]. The spheroids were grown in these Matrigel droplets, and cells were visualized by their expression of the nuclear protein histone 2B, fused to GFP. A small-scale RNAi screen was then carried out, allowing the researchers to identify genes associated with mitosis, detected by imaging using a dual-view inverted selective plane illumination microscope (SPIM). Image analysis included volumetric measurements of not only the entire spheroid but also the individual nuclei within each cell, providing a high level of detail with respect to the changes seen on RNAi treatment. While this particular study did not analyze components of the endomembrane system, it does nevertheless demonstrate feasibility of the approach, and in particular capability of generating highly uniform spheroids for volumetric analysis with subcellular resolution.

Uniformity of spheroid growth is one important characteristic not only from a screening perspective but also because it simplifies downstream analysis pipelines. One method to achieve this is through the use of geometrical constraints coupled to microfluidics (Figure 1G). One such example has been reported in which up to 500 spheroids can be grown on a single chip [47]. The cells were prepared in a droplet of medium and liquid agarose and were caught by hexagonal ‘anchors’ as they flowed through the device. Spheroid formation occurred within 24 h, and they showed a high degree of uniformity in size and shape. Long-term growth was achieved by exchange of cell culture medium through the device. In this initial work, they were able to measure the organization of the cells in spheroids, culture different types of cells on the same chip, as well as selectively recover spheroids of interest using local heating by an infrared laser beam [47]. This approach was further developed by addition of a second anchor to the primary anchors. These secondary anchors had a lower trapping force than the primary anchors, and allowed the use of two populations of droplets. The first population contained the cells to form the spheroids and the second population of droplets was used to manipulate the spheroids, for example allowing establishment of co-cultures or treatment with drug libraries [48]. This device has also been used to study the formation of organoids by fluorescence imaging [49], and to study the secretion of vascular endothelial growth factor by mesenchymal stem cell spheroids [50]. These studies clearly show the potential of this method to grow and manipulate spheroids, and applications for study of the endomembrane system in spheroids grown in such a device should be extensive. It is important to note however, that analysis of spheroids in such devices remains challenging, and so far these studies reported have not applied volumetric analysis to the spheroids, but rather single plane analysis. Potentially, such devices could be coupled to a light-sheet microscope, which has the specific advantage of being able to rapidly image in multiple planes. Others have shown that such a set-up can be used to image flowing single cells in suspension, as well as small clusters of cells with subcellular resolution. Subsequent volumetric analysis was used to measure, for example, the distribution and size of organelles [51]. Although this study did not use spheroids, one could envisage that this would be possible, and thereby combining the manipulation abilities of microfluidic devices with this imaging approach would be a very powerful method to study the endomembrane system in a 3D assembly.

**Figure 2:**
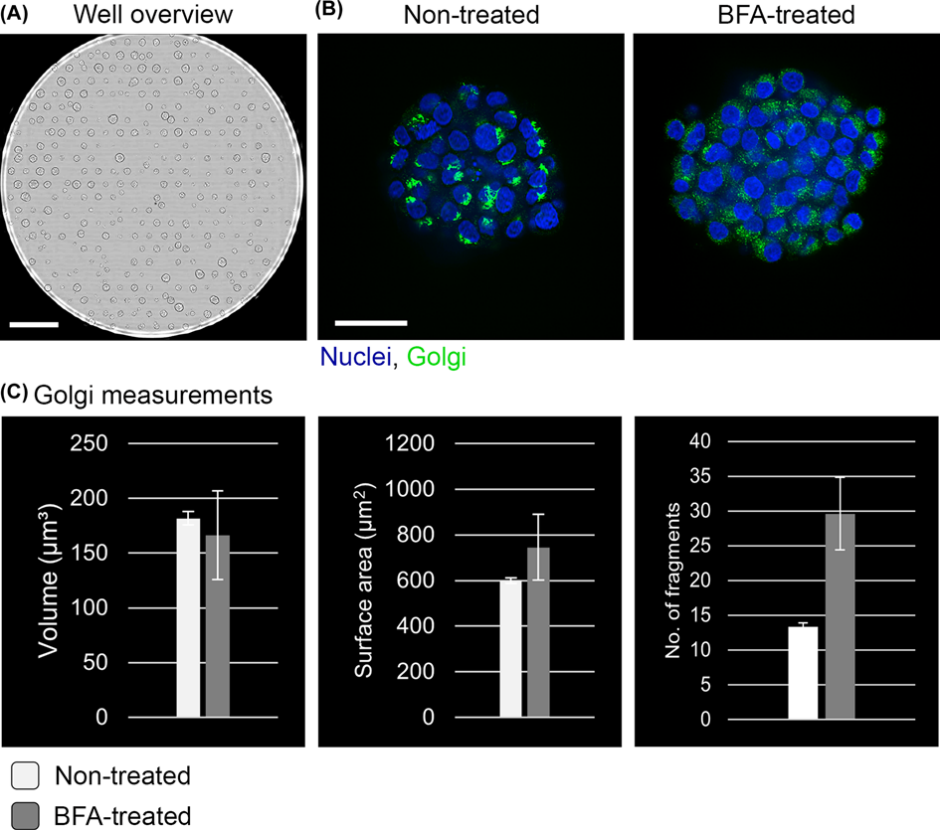
Morphological analysis of the Golgi apparatus in spheroids Example of HeLa cell spheroids grown on micropatterns in the well of a 96-well plate, followed by their automated imaging and analysis. (**A**) Bright-field image of an entire well showing ordered arrangement of the spheroids after 3 days of growth. Scale bar represents 1 mm. (**B**) Single confocal plane fluorescence image of an individual spheroid on the micropattern. The image on the left is of a control (untreated) spheroid, the image on the right is of a spheroid treated for 20 min with Brefeldin A (BFA). Nuclei stained with Hoechst 33342 are in blue, Golgi apparatus immunostained for the Golgi matrix protein GM130 are in green. Scale bar represents 50 µm. (**C**) Examples of volumetric analysis performed on the Golgi apparatus in individual cells within untreated and BFA-treated spheroids. Parameters of Golgi volume, Golgi surface area, and number of Golgi fragments are shown. Data come from 10 independent spheroids. Data are from the author’s own laboratory.

Very early in the utilization of high-content screening approaches, researchers realized that there can be high morphological variability between the individual cells found in a monolayer. As such, methods to grow individual cells in a constrained manner and in a specific shape emerged, particularly employing micropatterning technology. Two landmark studies grew individual monolayer cells on these micropatterns and showed that cells organize themselves differently in cell division and interphase when compared with cells that are not grown in a confined manner [52,53]. More recently, this method was further developed to grow spheroids of a uniform size and shape, as well as in defined positions. Several different cell lines (HT-29, HCT-116, T-47D, MCF-7, MDA-MB-231, A549 and HeLa cells) were grown as spheroids on these micropatterns, and alongside assessing cell death using propidium iodide staining, they measured spheroid diameter, spheroid roundness, and fluorescence levels at ether the center or edge of the spheroid. They proposed that this system was ideal for fully automated high-content screening [54]. This method of growing spheroids has the advantage that spheroids are uniform in size and shape and are arranged in defined rather than random positions within a well of a multi-well plate (Figure 1E). This latter point thereby reduces the need for imaging an excessive area of a well, which in turn has a benefit in reducing the volume of data acquired. By adjusting the size of each micropattern, as well as the pitch between each pattern, highly regular arrays of several hundred spheroids can be generated in a single well of a 96-well plate (Figure 2A). Micropatterning on glass-bottomed multi-well plates opens up the potential for using confocal high-content screening microscopy to image these spheroids with subcellular resolution, revealing the morphology of organelles such as the Golgi apparatus in each of the cells in the spheroid (Figure 2B). This in turn allows cell-based assays to be carried out, for example treatment of the spheroids with classical endomembrane perturbation drugs, such as brefeldin A (BFA), which have proved to be highly useful tools for our deepening our understanding of the endomembrane system [55]. Analysis of such images, at subcellular resolution level, provide the opportunity to quantify features such as organelle volume, surface area and number of fragments (Figure 2C).

Another similar approach to surface micropatterning is the use of microwells (Figure 1H). These can be produced in a variety of ways, using materials ranging from agarose to poly-dimethylsiloxane (PDMS). In some cases these microwells can have ECM materials such as collagen added, allowing the spheroids to become completely encapsulated. One study used this technique to investigate hypoxia-induced changes in invasion, proliferation, and phenotypic transition in glioma spheroids [56]. Another study used two different hydrogels, namely polyethylene glycol (PEG) and calcium alginate, in a co-culture model to demonstrate the importance of secretory factors to induce stem cell differentiation. The innovation in this set-up was that the PEG hydrogel solidified when exposed to light and the alginate hydrogel could be chemically dissolved, thereby creating a mechanism for generating an alternating micropattern of columns of the two different hydrogels, each containing the different cell types [57]. More recently, similar approaches have been miniaturized still further, down to the use of 384-well plate formats, with the cell assemblies used as micro-tumors in drug screening studies [58]. Although these latter approaches did not acquire single cell resolution data from the spheroids, these methods of micropatterning and microwell generation display the potential for use in a wider set of cell-based assays. If problems of light penetration and scattering can be overcome, imaging methods could be applied.

## Volumetric image analysis

The final major step once suitable image data have been acquired is to maximize the quantitative information that can be extracted from the 3D assembly, ideally at single cell and subcellular resolution. Unfortunately, the vast majority of studies to date have chosen to analyze single and/or selected confocal planes rather than using volumetric analysis. The field of volumetric image analysis is still one that is very much under development. While there are a small number of commercial software platforms that have this functionality, these are expensive, typically require high computational power, and are not designed to process multicellular spheroid or organoid data on a large scale. However, new open-source tools are beginning to emerge. Recently, Piccinini and colleagues compared several open-source software tools/algorithms for volumetric analysis of nuclei, highlighting their strengths and weaknesses [59]. What is perhaps surprising from this study is that despite cell nuclei being perhaps one of the simplest organelles to identify, segment and measure (given their relatively standard ovoid shape), the tools tested all performed very differently. This implies that analysis of more pleomorphic subcellular structures, as found in the endomembrane system, will be even more challenging to extract quantitative volumetric data from (Figure 2C). Other studies have made volumetric measurements in spheroids by segmenting cells and organelles on each confocal *z*-plane and then integrating the voxels of the respective regions [60]. Again, probably the greatest success using this approach has been seen in attempts to measure phenotypes of nuclei. One study measured the deformation of nuclei in spheroids by measuring the voxel number, highlighting that an increase in spheroid size (and therefore in number of cells), increases the pressure on nuclei, and that the distribution of this stress on nuclei differs according to the shape of the spheroids [61]. Nuclei shape and orientation in spheroids grown in scaffold-free and scaffold-based systems have also been examined by light-sheet microscopy. In this case the analysis was performed using the ‘Fit Ellipsoid’ plugin of the open source image processing software, Icy [62]. Light-sheet microscopy seems to be an appropriate imaging tool to generate such data for volumetric measurements, potentially allowing as many as 30 features from individual nuclei in spheroids to be extracted [63]. It should be noted, however, that there are significant challenges with respect to the handling, storage and analysis of the large imaging datasets that are acquired when working with 3D cell assemblies. Both high-content screening microscopy and light-sheet microscopy can create terabytes of data within a few hours of imaging. Appropriate data handling pipelines and solutions are emerging, but it is likely that this challenge will remain with us [64,65]. Taken together, these examples highlight that volumetric analysis is making progress, and will undoubtedly shed more light on the mechanics of how cells behave within a 3D assembly. More work will be needed to develop algorithms that have improved ability to detect, segment, and quantify the myriad of membrane organelles, often of irregular size and shape, that are found through the cell cytoplasm.

## Conclusions

In conclusion, while it is clear that our understanding of the endomembrane system in the context of the single (monolayer) cell is now well advanced, many open questions remain with respect to whether this functionality is the same when cells are grown in the confined environment of a 3D cell assembly. However, the tools to both grow such assemblies, and probe them in a quantitative manner by imaging are rapidly evolving, and it seems inevitable that the next few years will see a further emphasis on the use of these models in basic cell biology. Our vision of quantitatively describing the ‘cell^3^’ is perhaps closer than we might dare to believe!

## Abbreviations

BFA: Brefeldin A
ER: endoplasmic reticulum
GEF: guanine nucleotide exchange factor
MCTS: multicellular tumor spheroids
MDCK: Madin-Darby Canine Kidney
PDMS: poly-dimethylsiloxane
PEG: polyethylene glycol
SPIM: selective plane illumination microscope
